# Postoperative pulmonary vascular growth in patients with congenital diaphragmatic hernia

**DOI:** 10.1007/s00383-024-05706-z

**Published:** 2024-05-07

**Authors:** Jun Fujishiro, Rina Matsuda, Takuji Tomari, Akinori Ichinose, Kaori Morita, Shinya Takazawa, Mariko Yoshida

**Affiliations:** Department of Pediatric Surgery, Faculty of Medicine, The University of Tokyo, Hongo 7-3-1, Bunkyo-ku, Tokyo, 113-8655 Japan

**Keywords:** Congenital diaphragmatic hernia, Lung vascular growth, Thoracoscopic approach

## Abstract

**Background:**

Postoperative pulmonary growth in congenital diaphragmatic hernias (CDH) remains unclear. We investigated postoperative pulmonary vascular growth using serial lung perfusion scintigraphy in patients with CDH.

**Methods:**

Neonates with left CDH who underwent surgery and postoperative lung perfusion scintigraphy at our institution between 2001 and 2020 were included. Patient demographics, clinical courses, and lung scintigraphy data were retrospectively analyzed by reviewing medical records.

**Results:**

Twenty-one patients with CDH were included. Of these, 10 underwent serial lung scintigraphy. The ipsilateral perfusion rate and median age on the 1st and serial lung scintigraphy were 32% (34 days) and 33% (3.6 years), respectively. Gestational age at prenatal diagnosis (*p* = 0.02), alveolar–arterial oxygen difference (A-aDO_2_) at birth (*p* = 0.007), and preoperative nitric oxide (NO) use (*p* = 0.014) significantly correlated with the 1st lung scintigraphy. No other variables, including operative approach, were significantly correlated with the 1st or serial scintigraphy findings.

All patients improved lung perfusion with serial studies [Difference:  + 7.0 (4.3–13.25) %, *p* = 0.001, paired *t*-test]. This improvement was not significantly correlated with preoperative A-aDO_2_ (*p* = 0.96), NO use (*p* = 0.28), or liver up (*p* = 0.90). The difference was significantly larger in patients who underwent thoracoscopic repair than in those who underwent open abdominal repair [+ 10.6 (5.0–17.1) % vs. + 4.25 (1.2–7.9) %, *p* = 0.042].

**Conclusion:**

Our study indicated a postoperative improvement in ipsilateral lung vascular growth, which is possibly enhanced by a minimally invasive approach, in patients with CDH.

## Highlights

- Whether the postoperative ipsilateral lung perfusion improves or worsens in patients with CDH remains unknown. Factors associated with the potential lung growth have not yet been fully investigated.
- Our study indicated a postoperative improvement in ipsilateral lung vascular growth, which is possibly enhanced by a minimally invasive approach in patients with CDH.

## Introduction

The survival of patients with congenital diaphragmatic hernia (CDH) has recently improved due to modern treatment strategies, including gentle ventilation with permissive hypercapnia and delayed surgical repair after stabilization [1]. Conflicting reports exist on postoperative lung vascular growth and function after CDH repair [2–6]. Arena et al. reported rapid improvement in lung perfusion after CDH repair, even in apparently hypoplastic and small lungs in infant, adolescent, and adult patients with CDH [4]. Another single-center study using multiple lung perfusion scintigraphy concluded that the majority of survivors showing significant vascular growth had symptom-free lives [5]. However, another study in a cohort of CDH survivors with more severe disease characteristics showed deterioration of ventilation/perfusion (V/Q) mismatch due to perfusion deficits in the ipsilateral lung [6]. Furthermore, factors affecting postoperative lung perfusion have not been thoroughly investigated previously in the literature.

This study aimed to determine the possible vascular growth in the ipsilateral lung and the factors affecting it in CDH survivors using serial lung perfusion scintigraphy.

## Methods

Patients with left Bochdalek hernia, who were diagnosed prenatally or within 24 h after birth, underwent CDH repair during the neonatal period from January 1st, 2001 to December 31st, 2020, and who received at least 1 postoperative lung perfusion scintigraphy at our institution were included in the study. The following patient variables were retrospectively extracted from the medical records and used for analysis: sex, gestational age at prenatal diagnosis, gestational age at birth, birth weight, Apgar score (1 min, 5 min), alveolar–arterial oxygen difference (A-aDO_2_) at birth and immediately before the operation, nitric oxide (NO) use during preoperative stabilization and at the time of surgery, liver up, defect size (congenital diaphragmatic hernia study group (CDHSG) staging A–D) [7], direct/patch closure, operative approach (thoracoscopy/open abdominal), and age at discharge. Age and ipsilateral lung perfusion rate at the first and serial lung perfusion scintigraphy were also obtained. The results from the first and last perfusion studies were used if three or more studies were performed.

For lung perfusion scintigraphy, macroaggregated albumin particles tagged with 99 technetium, at a dose of 50,000–100,000 particles were slowly injected. Planar images were obtained from the anterior, posterior, and lateral projections. The percentage function of the individual lungs was calculated.

The primary outcome of the study was the difference between the 1st and serial lung perfusion study in patients with CDH. As secondary outcomes, we also analyzed the result of 1st and serial study. Categorical and numerical variables were expressed as *n* (%) and median (interquartile range), respectively. The Wilcoxon test, Pearson’s correlation coefficient test, and paired *t*-test were used for categorical, numeric, and paired variables, respectively. We used JMP14, (SAS Institute Inc., NC, USA) for statistical analysis. The correlation coefficient was expressed as *r*. Statistical significance was set at *p* < 0.05. This study was approved by the Institutional Review Board of the Research Ethics Committee of the Faculty of Medicine, the University of Tokyo [No.2996-(10)].

## Results

Twenty-one patients with CDH who underwent surgery during the neonatal period and at least one postoperative lung perfusion scintigraphy were included in the study. Non-isolated patients with CDH and severe associated anomalies, including chromosomal abnormalities and major cardiac malformations, were not included in the study. All 21 patients were diagnosed prenatally, had a left-sided hernia, and were free from extracorporeal membrane oxygenation (ECMO) during the perioperative course. NO therapy was used in seven (33%) patients for preoperative stabilization. The number of patients with each CDHSG defect size were: A, 8 (38.1%); B, 7 (33.3%); C, 3 (14.3%); and D, 3 (14.3%). Of the total, 12 and 9 patients underwent open abdominal repair (O) and thoracoscopic repair (T), respectively. Among these patients, 11 underwent one lung perfusion study and 10 underwent multiple studies. The patient characteristics were similar. However, patients in multiple studies underwent surgery at older ages (4.5 days vs. 2 days, *p* = 0.03) and showed lower ipsilateral lung perfusion (25.45% vs. 38%, *p* = 0.049) in the 1st study, suggesting that patients in multiple studies had severe disease (Table 1).

**Table 1:** Patient demographics

Variables	All patients	One study	Multiple studies	*p*
*n*	21	11 (52.3%)	10 (47.7%)	
Sex				1.00
Male	10 (47.7%)	5 (45.5%)	5 (50%)	
Female	11 (52.3%)	6 (54.5%)	5 (50%)	
Gestational age (weeks)	37 (37, 38)	38 (37, 39)	37 (35.75, 38)	0.07
Birth weight (g)	2705 (2424, 2994)	2862 (2637, 3422)	2542.5 (2360, 2979)	0.20
Apgar score				
At 1 min	2.5 (1.25, 5.75)	2.5 (2, 6.25)	2.5 (1, 5.5)	0.49
At 5 min	3 (3, 4)	4 (3, 4.5)	3 (2, 4)	0.09
A-aDO_2_ (mmHg)	261 (128, 546)	250.4 (130.5, 546)	344.6(118, 560)	1.00
NO use	7 (33%)	3 (27.3%)	4 (40%)	0.66
Age at operation (days)	3 (2, 5)	2 (2, 3)	4.5 (2.75, 5.5)	0.03
Liver up	7 (33.3%)	4 (36.4%)	3 (30%)	1.00
Patch closure	5 (23.8%)	3 (27.3%)	2 (20%)	1.00
CDHSG defect size				0.88
A	8 (38.1%)	4 (36.4%)	4 (40.0%)	
B	7 (33.3%)	4 (36.4%)	3 (30.0%)	
C	3 (14.3%)	1 (9.1%)	2 (20.0%)	
D	3 (14.3%)	2 (18.2%)	1 (10.0%)	
Operative approach				0.20
Open abdominal	12 (57.1%)	8 (72.7%)	4 (40%)	
Thoracoscopy	9 (42.9%)	3 (27.3%)	6 (60%)	
Age at discharge (days)	32 (25, 53.5)	30 (26, 51)	34 (23.75, 58.25)	0.72

*A-aDO*_*2*_ alveolar–arterial oxygen difference, *CDHSG* congenital diaphragmatic hernia study group, *NO* nitric oxide

**Fig. 1:**
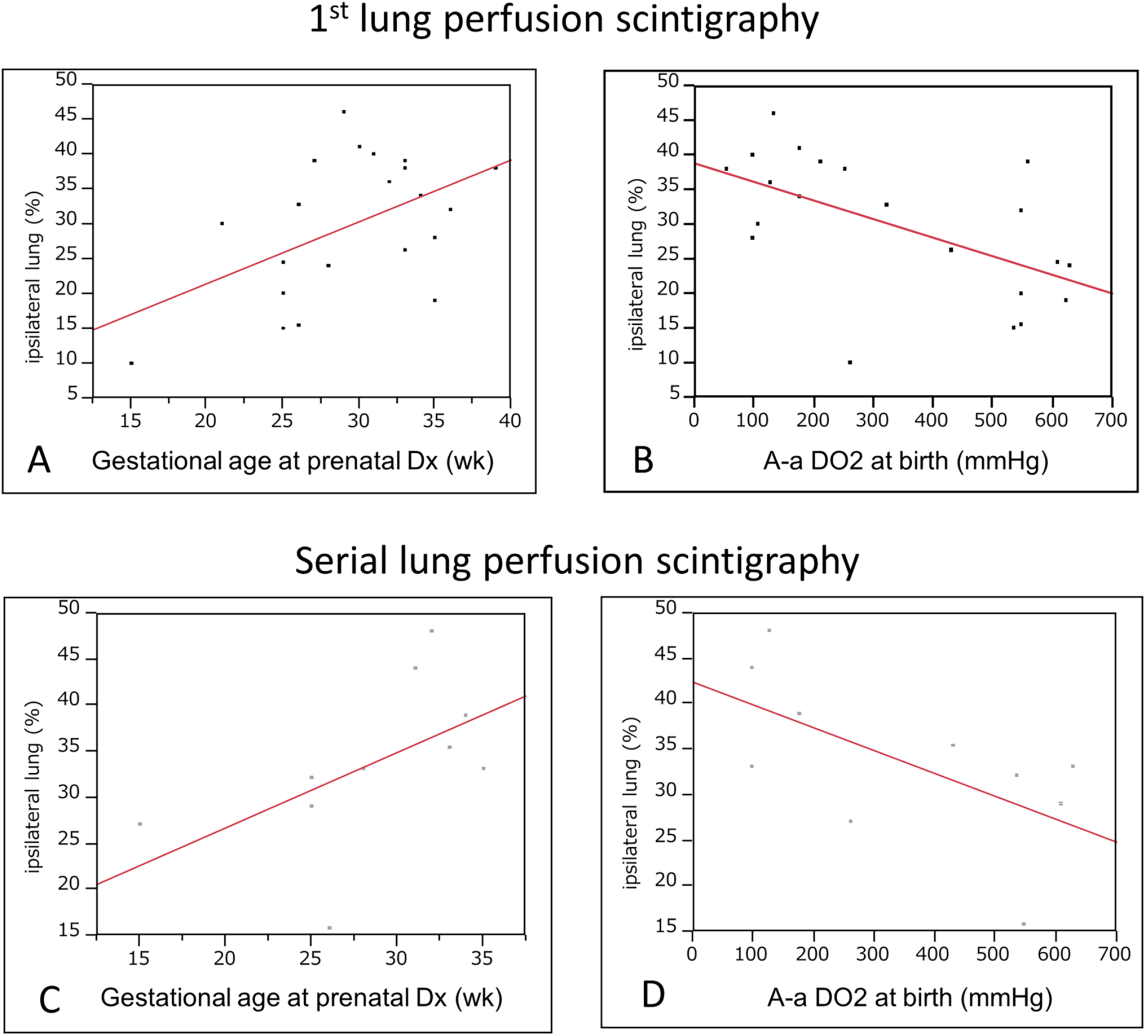
Ipsilateral lung perfusion after CDH repair. (**A**) Association between gestational age at prenatal diagnosis and the 1st perfusion study (*r* = 0.887, *p* = 0.02). (**B**) Association between A-aDO_2_ at birth and the 1st perfusion study (*r* =  − 0.027, *p* = 0.007). (**C**) Association between gestational age at prenatal diagnosis and the serial perfusion studies (*r* = 0.82, *p* = 0.10). (**D**) Association between A-aDO_2_ at birth and the serial perfusion studies (*r* =  − 0.025, *p* = 0.06). *A-aDO*_*2*_ alveolar–arterial oxygen difference

Among the 21 patients who underwent one or multiple perfusion studies, the median age and the ipsilateral perfusion rate was 34 (22, 44.25) days and 32 (22, 38.5) % in the first perfusion study, respectively (Table 2). Gestational age at prenatal diagnosis (*p* = 0.02), A-aDO_2_ at birth (*p* = 0.007), and preoperative NO use (*p* = 0.014, data not shown) significantly correlated with the 1st perfusion study (Fig. 1A, B). No other variables, including the operative approach (T 32.7 (20.7, 38.5) %; O 31 (21, 38.75) %, *p* = 0.943), were significantly associated with the 1st perfusion study (data not shown).

**Table 2:** Result of lung perfusion scintigraphy

	All patients	One study	Multiple studies	*p*
*n*	21	11	10	
1st lung perfusion study				
Age (days)	34 (22, 44.5)	35 (22, 45)	32 (22, 47.75)	0.80
Ipsilateral lung (%)	32 (22, 38.5)	38 (30, 45)	25.45 (15.5, 34.5)	0.049
Serial perfusion study				
Age (years)		–	3.6 (1.1, 10.2)	
Ipsilateral lung (%)		–	33 (28.8, 40.25)

The median age at the time of the serial studies was 3.6 (1.1, 10.2) years among 10 patients with multiple studies (Table 2). The ipsilateral perfusion rate in the serial studies was 33 (28.8, 40.25) %. While there was a tendency for higher perfusion rates in patients with older gestational age at prenatal diagnosis (*p* = 0.10, Fig. 1C) or smaller A-aDO_2_ at birth (*p* = 0.06, Fig. 1D), no perioperative parameters, including preoperative NO use (*p* = 0.24) and operative approach (*p* = 0.45), were significantly associated with the serial ipsilateral perfusion rate.

**Fig. 2:**
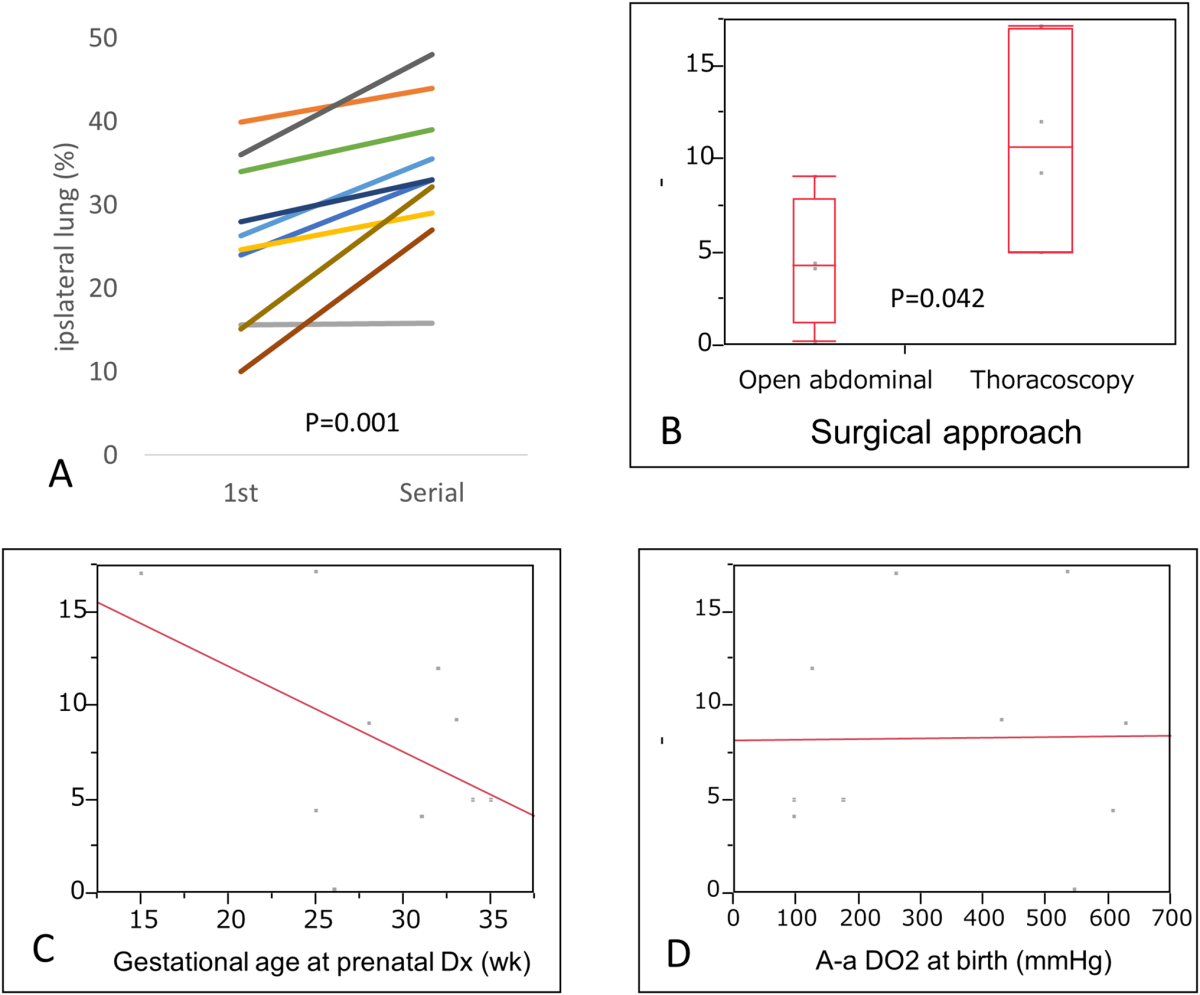
Differences between the 1st and serial perfusion studies. (**A**) Changes between the 1st and serial perfusion studies in each patient (*n* = 10, *p* = 0.001). (**B**) Differences between the 1st and serial perfusion studies in patients with open abdominal repair and thoracoscopic repair (Thoracoscopy 10.6 (5.0, 17.1) %; Open 4.25 (1.2, 7.9) %, *p* = 0.042). (**C**) Association between the difference in lung perfusion and gestational age at prenatal diagnosis (*r* =  − 0.456, *p* = 0.16). (**D**) Association between the difference in lung perfusion and A-aDO_2_ at birth (*r* = 0.0004, *p* = 0.97). *A-aDO*_*2*_ alveolar–arterial oxygen difference

When the 1st and the serial perfusion study were compared, perfusion rate increased with age in all patients (difference:  + 7 (4.3, 13.25) %, *p* = 0.001) (Fig. 2A). The difference was significantly larger in patients who underwent thoracoscopic repair than in those who underwent the open abdominal approach (T: 10.6 (5.0, 17.1) %; O: 4.25 (1.2, 7.9) %, *p* = 0.042) (Fig. 2B). This difference was not significantly associated with other variables, including gestational age at prenatal diagnosis (*p* = 0.16, Fig. 2C), A-aDO2 (*p* = 0.96, Fig. 2D), NO use (*p* = 0.28), liver up (*p* = 0.90), or patch closure (*p* = 0.08).

## Discussion

In this study, we investigated the postoperative status and natural history of ipsilateral lung perfusion in patients with CDH. Our cohort consisted of patients with relatively mild CDH, which was suggested by the smaller CDHSG defect size and the lack of need for ECMO. Gestational age at prenatal diagnosis, A-aDO2 at birth, and preoperative NO use significantly correlated with the 1st lung perfusion study at a median age of 34 days. All patients with serial perfusion studies showed significant improvement in ipsilateral lung perfusion (Fig. 2A). The thoracoscopic approach was significantly associated with a larger difference between the 1st and serial studies (Fig. 2B).

The natural history of the ipsilateral lung function is a matter of concern in patients undergoing CDH repair. Significant lung growth occurs at the alveolar level with postnatal vascular remodeling, resulting in larger and fewer muscular arteries after CDH repair [8], suggesting that the anatomical changes caused by CDH are, at least in part, reversible [8–10]. While some studies have shown persistent postoperative reduction of ipsilateral lung perfusion and impaired ventilation/perfusion (V/Q) mismatch [2, 3, 6, 11], other studies have suggested that lung perfusion improves postoperatively with age [4, 5]. Spirometry studies after CDH repair have also shown conflicting results, ranging from normal to obstructive, restrictive, and mixed dysfunction [12–14]. This inconsistency could be due to the differences in the populations and designs of each study. Recent progress in the management of CDH may have led to the differences between studies. However, even with the possible impairment of ventilation and perfusion functions in the ipsilateral lung, the majority of these studies demonstrated that cardiopulmonary symptoms were rarely observed in patients with CDH, especially long after surgery [12–14]. Similarly, all the patients in the present study were free of respiratory symptoms.

It is still controversial whether the postoperative ipsilateral lung perfusion improves, worsens or remains unchanged in patients with CDH. Previous studies with small cohorts demonstrated poor lung perfusion with limited follow-up periods [13, 15]. Other studies have suggested that factors related to severe diseases, such as liver up, patch closure, and ECMO usage [16, 17] are associated with poor perfusion. The results of our 1st perfusion study are consistent with those of previous studies. Gestational age at prenatal diagnosis, A-aDO2 at birth, and preoperative NO, all of which are indicators of CDH severity, were associated with the 1st perfusion study. A relatively large cohort from an American group showed that the V/Q mismatch worsened gradually in survivors of CDH, especially in those with severe disease characteristics, as a result of a decrease in ipsilateral lung perfusion [6]. Their cohort consisted of patients with severe disease, with most having a CDHSG defect size of C/D. In contrast, patients in studies showing improvement in ipsilateral lung perfusion with age were less severe, such as those without ECMO or patch closure [4, 5]. In our study, in which the majority of patients had a CDHSG defect size A/B with none requiring ECMO, all patients demonstrated improvement in ipsilateral lung perfusion with age, and the thoracoscopic approach was associated with a larger increase in perfusion. Considering the results of these studies, postoperative improvement in ipsilateral perfusion can occur in patients with mild CDH while it worsens with age in patients with severe CDH. This is consistent with a previous study that showed a continuous improvement in lung perfusion in a patient with modest CDH, but not in patients with severe CDH [5].

Our study implied that a minimally invasive approach may enhance improvement in patients with mild CDH. Due to the small number of patients and potential bias for the retrospective study, this finding might be a coincidence. However, our study showed no significant association between postoperative improvement in lung perfusion and factors associated with severity of CDH such as A-aDO_2_, NO use, liver up, or patch closure, thoracoscopic approach rather than severity of CDH would affect postoperative improvement in lung perfusion. A possible explanation is that limited damage to the respiratory system during thoracoscopic surgery may have contributed to this improvement. Previous studies have described possible increases in respiratory morbidity or functional derangement due to iatrogenic damage during the neonatal perioperative period [13, 18].

The limitations of our study include its retrospective design and small number of patients. This study is subject to selection bias resulting from a tendency for serial studies to be performed in patients with severe CDH indicated by lower ipsilateral lung perfusion. Although modern treatment strategies, including gentle ventilation and surgery after stabilization, were applied for CDH over the study period, a possible change in treatment for CDH at our institution during the study period might have affected the outcome. Due to the retrospective nature of the study, prenatal imaging and postoperative echocardiogram were not systematically performed in all cases, and data obtained from these analyses, including the observed to expected lung area to head circumference ratio (O/E LHR) and pulmonary artery diameter, were not available for the study. The adoption of the thoracoscopic approach during the study period is another potential source of bias. However, the open abdominal approach for patients with mild disease was included in this study with this study period. Most patients with mild disease have recently undergone thoracoscopic repair at our institution.

In conclusion, our study using postoperative lung perfusion scintigraphy demonstrated that vascular growth in the ipsilateral lung was impaired, and that the severity of impairment was associated with the severity of the disease in patients with CDH. Postoperative vascular growth in the ipsilateral lung occurs with age, at least in patients with mild disease, as observed in our cohort. The thoracoscopic approach might contribute to better vascular growth in the ipsilateral lungs. Further large-scale clinical and experimental studies are necessary to understand postoperative lung development in patients with CDH.

## Data Availability

The datasets generated and/or analyzed during the current study are available from the corresponding author on reasonable request.

## Abbreviations

A-aDO_2_: Alveolar–arterial oxygen difference
CDH: Congenital diaphragmatic hernia
CDHSG: Congenital diaphragmatic hernia study group
ECMO: Extracorporeal membrane oxygenation
NO: Nitric oxide
